# An Internet-based emotion regulation intervention versus no intervention for non-suicidal self-injury in adolescents: a statistical analysis plan for a feasibility randomised clinical trial

**DOI:** 10.1186/s13063-021-05406-2

**Published:** 2021-07-16

**Authors:** Markus Harboe Olsen, Britt Morthorst, Anne Katrine Pagsberg, Michella Heinrichsen, Bo Møhl, Lotte Rubæk, Johan Bjureberg, Olivia Simonsson, Jane Lindschou, Christian Gluud, Janus Christian Jakobsen

**Affiliations:** 1 Copenhagen Trial Unit, Centre for Clinical Intervention Research, Copenhagen University Hospital – Rigshospitalet, Copenhagen, Denmark; 2 Department of Neuroanaesthesiology, Neuroscience Centre, Copenhagen University Hospital – Rigshospitalet, Copenhagen, Denmark; 3 Research Unit, Child and Adolescent Mental Health Services, Copenhagen University Hospital - Herlev and Gentofte, Copenhagen, Denmark; 4 Department of Clinical Medicine, Faculty of Health, Copenhagen University, Copenhagen, Denmark; 5 Department of Communication and Psychology, Aalborg University, Aalborg, Denmark; 6 Team of Self-Injury, Child and Adolescent Mental Health Services, Copenhagen, Denmark; 7 Department of Psychology, Stanford University, Stanford, CA USA; 8 Centre for Psychiatry Research, Department of Clinical Neuroscience, Karolinska Institutet & Stockholm Health Care Services, Region Stockholm, Stockholm, Sweden; 9 Department of Regional Health Research, Faculty of Health Sciences, University of Southern Denmark, Odense, Denmark

**Keywords:** Non-suicidal self-injury, Emotion Regulation Individual Therapy for Adolescents (ERITA), Internet-based intervention, Randomised feasibility trial, Statistical analysis plan

## Abstract

**Background:**

Non-suicidal self-injury (NSSI) has a lifetime prevalence of 17% in adolescents in the general population and up to 74% in adolescents with psychiatric disorders. NSSI is one of the most important predictors of later suicidal behaviour and death by suicide. The TEENS feasibility trial was initiated to assess the feasibility and safety of Internet-based Emotion Regulation Individual Therapy for Adolescents (ERITA) as an add-on to treatment as usual in 13–17-year-old patients with NSSI referred to the Child and Adolescent Mental Health Services.

**Methods:**

The TEENS feasibility trial is a randomised clinical trial with a parallel-group design. The trial intervention is an 11-week online therapy which is tested as an add-on to treatment as usual versus treatment as usual. The primary feasibility outcomes are the fraction of participants who (1) completed 12 weeks of follow-up interview or assessment, (2) consented to inclusion and randomisation out of all eligible participants, and (3) were compliant with the experimental intervention, assessed as completion of at least six out of eleven modules in the programme. Since this is a feasibility trial, we did not predefine a required sample size. The exploratory clinical outcome, the frequency of NSSI episodes, assessed using Deliberate Self-Harm Inventory – Youth version (DSHI-Y), at the end of intervention, is planned to be the future primary outcome in a larger pragmatic definitive randomised clinical trial. After completion of the feasibility trial, blinded data will be analysed by two independent statisticians blinded to the intervention, where ‘A’ and ‘B’ refer to the two groups. A third party will compare these reports, and discrepancies will be discussed. The statistical report with the analyses chosen for the manuscript is being tracked using a version control system, and both statistical reports will be published as a supplementary material. Based on the final statistical report, two blinded conclusions will be drawn by the steering group.

**Discussion:**

We present a pre-defined statistical analysis plan for the TEENS feasibility trial, which limits bias, p-hacking, data-driven interpretations. This statistical analysis plan is accompanied by a pre-programmed version-controlled statistical report with simulated data, which increases transparency and reproducibility.

**Trial registration:**

ClinicalTrials.govNCT04243603. Registered on 28 January 2020

**Supplementary Information:**

The online version contains supplementary material available at 10.1186/s13063-021-05406-2.

## Introduction

Non-suicidal self-injury (NSSI) has a lifetime prevalence of 17% in adolescents in the general population and up to 74% in adolescents with psychiatric disorders [1, 2]. NSSI is one of the most important predictors of later suicidal behaviour and death by suicide [2–5]. The *treatment as usual* for NSSI includes a variety of clinical treatments and assessments offered by the Child and Adolescent Mental Health Services (CAMHS). Treatment of NSSI is heterogeneous, with different interventions such as pharmacological, family-based, and cognitive-behavioural therapy (CBT); supportive counselling; and psychoeducation depending on the patients’ primary mental health problem [6–8].

No treatment has been found superior for NSSI in adolescents [9]. The stigmatisation of NSSI may lower the tendency to seek help and adhere to treatment, while Internet-based interventions are assumed to be more easily accepted [10–13]. Internet-based interventions for adolescents guided by a therapist are effective for several psychiatric disorders [14]. Only one previous feasibility study has investigated the potential of an Internet-based intervention (Emotion Regulation Individual Therapy for Adolescents (ERITA)) for NSSI in youth; this study found promising trends towards reduced NSSI frequency at the end of treatment provision; however, the results should be interpreted with caution based on single-arm design [10, 15, 16]. There is a need for randomised clinical trials assessing the effect of specialised Internet-based interventions for NSSI [17].

The TEENS feasibility trial was initiated to assess the feasibility and safety of Internet-based ERITA as an add-on to treatment as usual in 13–17-year-old patients with NSSI referred to the Child and Adolescent Mental Health Services [18]. This paper describes the plan for statistical analyses of the feasibility and exploratory clinical outcomes in the TEENS feasibility trial [18].

## Methods

The TEENS feasibility trial is a randomised feasibility trial with a parallel-group design [18]. The trial methodology in general has been described previously [18]. Patients are recruited from the Child and Adolescent Mental Health Services, the Capital Region, Denmark. The experimental intervention is therapist-guided Internet-based ERITA as an add-on to treatment as usual [10, 11, 18]. A detailed description of the Internet-based ERITA intervention can be found elsewhere [11]. Treatment as usual is provided by multidisciplinary teams in nine outpatient clinics within the Child and Adolescent Mental Health Services in the Capital Region of Denmark. The trial was registered on ClinicalTrials.gov (identification no. NCT04243603) before the inclusion of the first participant. Consent for inclusion and randomisation is carried out only if a patient fulfils all inclusion criteria and none of the exclusion criteria. The study design was based on the CONSORT extension for randomised pilot and feasibility trials [19], and our statistical analysis plan is based on the recommendation from Gamble et al. [20].

### Inclusion criteria

- Age 13 to 17 years, both inclusive
- ≥ 5 NSSI episodes during the past year and ≥ 1 NSSI episodes during the past month assessed by the Deliberate Self-Harm Inventory, Youth version (DSHI-Y) [21]
- Age-appropriate Danish literacy assessed by referring clinicians and the self-injury team
- At least one parent committing to participate in the parent programme
- Informed consent from parents or legal caretakers
- Informed consent from the participant above 15 years of age

### Exclusion criteria

- Elevated or imminent suicidal risk assessed by clinicians during routine screening (that can be rated as no risk, elevated risk, or imminent risk). In the latter two cases, the patient needs close supervision and possibly hospitalisation.

### Randomisation and blinding

Randomisation is, after informed consent, performed by a member of the self-injury team using the central web-based randomisation system managed by the Copenhagen Trial Unit (CTU, Copenhagen, Denmark). As this is a feasibility trial, we will not stratify the randomisation. Due to the nature of the intervention, blinding of participants and clinicians is not possible. The researchers are, however, blinded to the allocation. During the final phone and video interviews, the participants are instructed not to disclose the allocation outcome.

### Trial interventions

The trial intervention is an add-on to treatment as usual and consists of 11-week, manualised online therapy based on the methods of CBT, dialectical behaviour therapy, and acceptance and commitment therapy, the so-called ERITA [10, 11]. The programme consists of 11 modules following an initial introduction. The intervention also provides six modules for parents’ involvement focusing on the information about NSSI and other destructive behaviours, emotional awareness, effective communication skills (e.g. validation), and strategies to cope with their child’s negative emotions in an appropriate way. ERITA is provided online, meaning that the adolescents and the parents have online contact with an assigned clinical therapist during the 11-week intervention period. The adolescents are expected to complete a new module every week, i.e. eleven modules, while the parents must complete a module every second week, i.e. six modules. In addition, they are encouraged to review the youth modules every week. Through the course of the modules, the therapist will review the participant’s responses and provide written feedback through the Internet platform.

### Outcomes

The primary feasibility outcomes are the fraction of all participants who (1) completed 12 weeks of follow-up interview or assessment, (2) consented and were randomised of all eligible patients, and (3) were compliant with the experimental intervention, assessed as completion of at least six out of eleven modules in the programme and their parents completed at least 3 modules out of 6. Furthermore, several exploratory clinical outcomes are assessed (Table 1).

**Table 1:** Outcomes of the TEENS feasibility trial

Outcomes	Type of data
**Feasibility outcomes**	
Fraction of at least one completed clinical outcome (NSSI events) at end of the intervention	Proportion
Fraction of participants to include and randomise	Proportion
Fraction of compliance	Proportion
**Explorative primary clinical outcome**	
Frequency of non-suicidal self-injury *Deliberate Self-Harm Inventory (DSHI-Y)* [21]	Count; longitudinal
**Explorative secondary clinical outcomes**	
Quality of life *Kidscreen-10* [22]	Continuous; longitudinal
Symptoms of depression *Depression Anxiety Stress Scale (DASS-21)* [23]	Count; longitudinal
Symptoms of anxiety *Depression Anxiety Stress Scale (DASS-21)* [23]	Count; longitudinal
Symptoms of stress *Depression Anxiety Stress Scale (DASS-21)* [23]	Count; longitudinal
Self-injury *Yes/no*	Discrete; once
Sick days *Proportion of sick days during the last month*	Continuous; longitudinal
**Further explorative clinical outcomes**	
Difficulties in emotion regulation *Difficulties in Emotion Regulation Scale (DERS-16)* [24]	Continuous; longitudinal
Indirect self-destructive behaviours *Borderline Symptom List (BSL-supplement) + 1 item C-SSRS (ideations)*	Count; longitudinal
Suicidal ideations, plan, and actions *Columbia (C-SSRS)* [25]	^a^
Distress reactions—adolescent rated parents’ ability to cope with children’s negative emotions. *The Coping with Children’s Negative Emotions Scale (CCNES-APP)* [26]	Continuous; once
Punitive reactions—adolescent rated parents’ ability to cope with children’s negative emotions. *The Coping with Children’s Negative Emotions Scale (CCNES-APP)* [26]	Continuous; once
Expressive encouragement—adolescent rated parents’ ability to cope with children’s negative emotions. *The Coping with Children’s Negative Emotions Scale (CCNES-APP)* [26]	Continuous; once
Emotion-focused reactions—adolescent rated parents’ ability to cope with children’s negative emotions. *The Coping with Children’s Negative Emotions Scale (CCNES-APP)* [26]	Continuous; once
Problem-focused reactions—adolescent rated parents’ ability to cope with children’s negative emotions. *The Coping with Children’s Negative Emotions Scale (CCNES-APP)* [26]	Continuous; once
Minimization reactions—adolescent rated parents’ ability to cope with children’s negative emotions. *The Coping with Children’s Negative Emotions Scale (CCNES-APP)* [26]	Continuous; once
Adverse events *Negative Effects Questionnaire (NEQ)* [27]	Continuous; once

Longitudinal refers to the outcomes assessed at baseline and follow-up

^a^C-SSRS will be analysed at a later time point, as no consensus on methodology has been identified

### Sample size and power justification

Since this is a feasibility trial, we have not predefined a required sample size. The exploratory clinical outcome, the frequency of self-injury episodes, assessed using DSHI-Y [21], at the end of the intervention, is planned to be the primary future outcome in a larger pragmatic clinical trial. We plan to take the results of the exploratory clinical outcomes into consideration when estimating the required sample size and power estimation of both the primary and non-primary outcomes for the planned pragmatic randomised clinical trial. We have pragmatically chosen to include 15 participants in each group to provide acceptable robustness for the sample size calculation for a future larger pragmatic trial [19, 28] and to assess the proportion of missing data we could expect. Based on a reference of 13% missingness, this trial with the inclusion of 30 participants would be able to, by using a proportion power calculation for the binomial distribution with a power of 80% and an alpha of 0.05, dismiss an expected missingness of more than 32% in a future larger pragmatic trial.

### General analysis principles

Statistical analyses will be handled using R version 4.0.3 (R Core Team, Vienna, Austria) and Stata (StataCop LLC, TX, USA). All randomised participants will be included in all analyses. The baseline characteristics will be presented for each group (Table 2). For nationality, Danish citizens will be presented in their own group, and participants from other countries will be presented based on cultural properties, e.g. other European/North American or Middle Eastern. The threshold for significance in all analyses will be below 0.05 and will not be corrected for multiple comparisons, as the clinical outcomes are all exploratory and will be interpreted as such.

**Table 2:** Baseline characteristics based on simulated data

	A	B	Overall
n	15	15	30
Age (mean (SD))	16.13 (0.83)	15.64 (0.50)	15.90 (0.72)
Gender (%)			
Female	6 (40.0)	5 (33.3)	11 (36.7)
Male	3 (20.0)	2 (13.3)	5 (16.7)
Others	3 (20.0)	3 (20.0)	6 (20.0)
Transgender	3 (20.0)	5 (33.3)	8 (26.7)
Nationality (%)			
Danish	4 (26.7)	6 (40.0)	10 (33.3)
Other European/North American	3 (20.0)	2 (13.3)	5 (16.7)
Middle Eastern	3 (20.0)	6 (40.0)	9 (30.0)
Others	5 (33.3)	1 (6.7)	6 (20.0)
School (%)			
Boarding school	1 (6.7)	4 (26.7)	5 (16.7)
High school	3 (20.0)	3 (20.0)	6 (20.0)
Middle school	6 (40.0)	3 (20.0)	9 (30.0)
No school	2 (13.3)	2 (13.3)	4 (13.3)
Others	3 (20.0)	3 (20.0)	6 (20.0)
Parental status (%)			
Cohabitant	3 (20.0)	3 (20.0)	6 (20.0)
Divorced	5 (33.3)	5 (33.3)	10 (33.3)
Married	4 (26.7)	2 (13.3)	6 (20.0)
Others	3 (20.0)	5 (33.3)	8 (26.7)

Missing data: age, 3.3%

### Statistical analysis

#### Analysis of primary feasibility outcomes

**Fig. 1:**
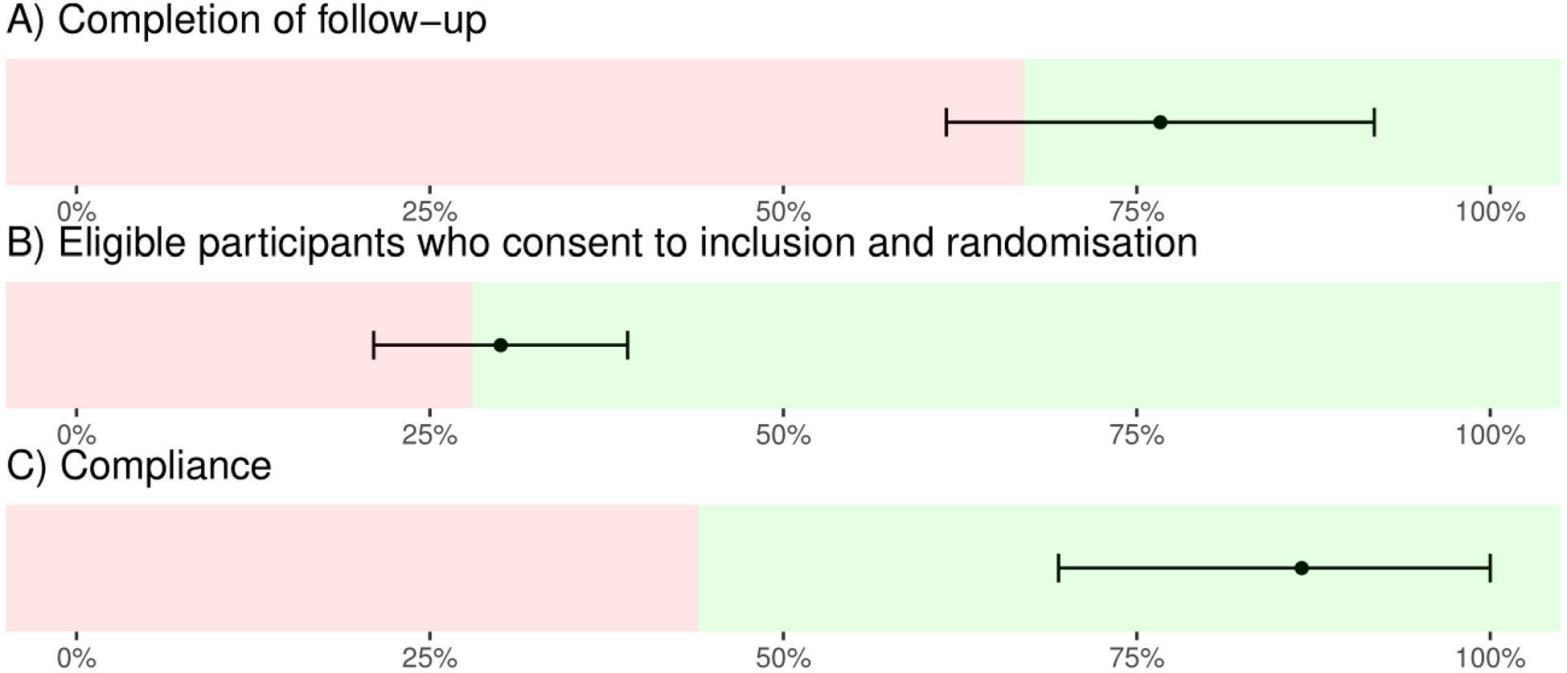
The presentation of the primary feasibility outcomes with colour-coding for every outcome, either feasible (green) or unfeasible (red). The lines presented in the figure are based on simulated data to exemplify the results presented in the final manuscript (supplemental material)

The feasibility outcomes were decided based on consensus and agreement between the investigators and were based on clinical expertise (Morthorst B, Pagsberg AK, Møhl B, and Rubæk L) and trial experience from previous pragmatic and feasibility trials (Lindschou J, Gluud C, and Jakobsen JC). These are all seen as relevant for carrying out a definitive large-scale trial. The primary feasibility outcomes are all based on the fraction of participants who fulfil our predefined criteria (see the ‘Inclusion criteria’ section). The fractions will be presented together with the confidence intervals using a 1-sample proportions test with continuity correction, with an adjusted maximum confidence limit of 100% (Fig. 1). The trial will be deemed feasible if (1) ≥ 87% (95% confidence intervals (95% CI) 68 to 96%) of participants completed at least one clinical outcome assessment (NSSI events) at the end of the intervention, (2) ≥ 40% (95% CI 29 to 52%) of all eligible patients were randomised, and (3) ≥ 73% (95% CI 45 to 91%) of the participants completed at least 6 modules out of 11 and their parents completed at least 3 modules out of 6. We have, after publishing the protocol [18], changed the statistical analyses for calculating the confidence intervals for proportions. The estimate of the lower confidence interval, using 1-sample proportions test with continuity correction, will serve as the least feasible indicator to conduct a large-scale trial. For instance, a lower confidence limit of 68% from the analysis will be interpreted as a feasible estimate for the first outcome.

#### Analysis of exploratory clinical outcomes

We plan to analyse the exploratory clinical outcomes as we plan to analyse these outcomes in the planned larger pragmatic trial, by choosing the analyses which fulfils the assumptions. The results will be interpreted with caution as this trial is not powered to investigate clinical outcomes, but the signals will help inform which outcomes we might choose for the larger pragmatic trial.

##### Continuous outcomes

Continuous exploratory clinical outcomes will be presented as means and standard deviations (SD) for each group, with an annotation in the tables of the percentage of missing data per group (Fig. 1A; Table 3). As previously recommended, we will use linear regression analyses adjusted for the baseline value for the continuous exploratory clinical outcomes [29].

**Table 3:** Summarised results of an exploratory outcome based on simulated data

	A	B	Overall
n	15	15	30
Kidscreen-10 (baseline) (mean (SD))	2.33 (1.43)	2.24 (0.86)	2.29 (1.16)
Kidscreen-10 (follow-up) (mean (SD))	− 2.06 (1.33)	− 2.02 (1.23)	− 2.04 (1.26)
Kidscreen-10 T-values (baseline) (mean (SD))	61.04 (14.37)	60.02 (8.35)	60.51 (11.44)
Kidscreen-10 T-values (follow-up) (mean (SD))	18.42 (13.38)	18.76 (11.87)	18.59 (12.39)

Missing data: Kidscreen-10 T-values (baseline), 3.3%; Kidscreen-10 T-values (follow-up), 3.3%

##### Count data outcomes

**Fig. 2:**
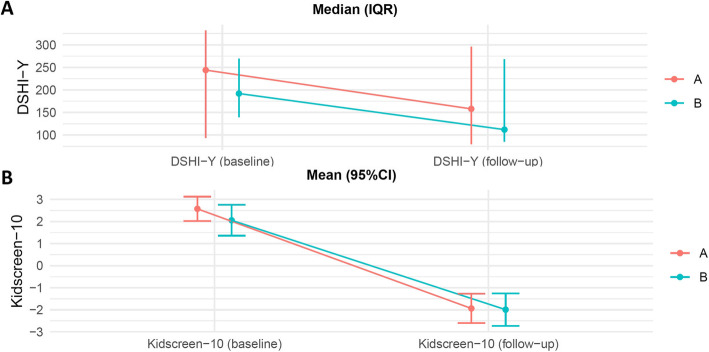
Graphical presentation of outcomes, which will be presented with either **A** median and IQR or **B** mean and 95% confidence interval

Count data exploratory clinical outcomes will be presented as medians and interquartile ranges for each group, with an annotation in the tables of the percentage of missing data per group (Fig. 2B). Count data exploratory clinical outcomes will be analysed using the Mann-Whitney U test. Hodges-Lehmann confidence intervals will be presented to demonstrate the uncertainty of the results [30].

##### Dichotomous outcomes

Dichotomous exploratory clinical outcomes will be presented as proportions for each group with an annotation in the tables of the percentage of missing data per group. Dichotomous exploratory clinical outcomes will be analysed using logistic regression. We will estimate the marginal effects to obtain RRs and confidence intervals of the RRs (based on ‘nlcom’ from Stata (StataCorp LLC, TX, USA)).

#### Handling of missing data

No specific methodology, including multiple imputations, will be used to handle missing data, but missingness will be listed in detail in the tables in the statistical reports (see below) as a tool to adapt the design of a larger pragmatic randomised trial.

#### Assessments of underlying statistical assumptions

The chosen analyses have few assumptions, with the main assumptions being related to the linear and logistic regressions [29, 31]. The variables included in the *linear regression models* will be visually assessed for normal distribution using histograms and quantile-quantile plots of the residuals and for homogeneity using residuals plotted against covariates and fitted values, with the possibility of a logarithmic transformation or applying robust standard errors to minimise deviations from the model [29].

The deviance divided by the degrees of freedom for *logistic regression model* will be calculated to assess relevant overdispersion. The logistic regression used will be univariable, i.e. with no covariates, and if few or zero events are identified (substantially lower than the rule of thumb of 10 events), the analyses will be carried out using Fisher’s exact test**.** The robustness of the confidence intervals and *p*-values might be affected by the small sample size, and these will be interpreted with caution [29].

### Statistical reports

A pre-programmed statistical report based on simulated data is publicly available on Zenodo (https://zenodo.org/record/4643529; European Organization for Nuclear Research, Genevé, Switzerland) and submitted as a supplemental material. After completion of the trial, blinded data will be analysed by two independent statisticians blinded to the intervention, where ‘A’ and ‘B’ refer to the two groups. The two statisticians will independently analyse all data and present the results in two independent reports. The two independent reports will be based on the agreed-upon pre-programmed statistical report. The coordinating investigator, the two statisticians and the Steering Committee will compare these reports and discrepancies will be discussed. The statistical report with consensus on the definitive analyses in the manuscript is being tracked using a version control system (https://github.com/lilleoel/CTU_TEENS, GitHub, San Francisco, CA, USA), and both statistical reports will, furthermore, be published as a supplementary material. Based on the final statistical report, two blinded conclusions will be drawn by the Steering Committee: one assuming ‘A’ is the experimental group and ‘B’ is the control group and one assuming the opposite. These abstracts will utilise the results from the blinded reports, and when the blinding is broken, the ‘correct’ abstract will be chosen and the conclusions in this abstract will not be revised. This described the process of analysing data, and interpreting data will also be used in the future large randomised clinical trial.

## Results

Not applicable

## Discussion

We present a detailed predefined description of the statistical analysis of the TEENS feasibility trial. The primary aim of this statistical analysis plan is to limit bias, p-hacking, and data-driven interpretations.

### Strengths

The primary strengths are the predefined statistical analysis plan and publication of a version-controlled pre-programmed statistical report before any data were available. This secures methodological transparency and enables the reproducibility of our results. Completion of a feasibility trial with three independent feasibility outcomes and multiple exploratory clinical outcomes will contribute with important data for the future randomised clinical trial we have planned.

### Limitations

Since no correction for multiplicity will be applied to the exploratory outcomes, any significance must be interpreted with caution. We assess multiple outcomes which increase the risk of false-positive results (type I error); any difference between the groups might be explained by random errors (‘play of chance’).

## Conclusion

We present a pre-defined statistical analysis plan for the TEENS feasibility trial, which limits bias, p-hacking, and data-driven interpretations. This statistical analysis plan is, furthermore, accompanied by a pre-programmed version-controlled statistical report with simulated data, which increases transparency and reproducibility.

## Supplementary Information

**Additional file 1.**

## Data Availability

The datasets generated and/or analysed during the current study are available at https://zenodo.org/record/4643529 and https://github.com/lilleoel/CTU_TEENS**.**

## Abbreviations

BSL-supplement: Borderline Symptom List
CAMHS: Child and Adolescent Mental Health Services
CBT: Cognitive behavioural therapy
CCNES-A: Coping with Children’s Negative Emotions Scale Adolescent
CCNES-APP: Coping with Children’s Negative Emotions Scale
C-SSRS: Columbia-Suicide Severity Rating Scale
CTU: Copenhagen Trial Unit
DASS-21: Depression Anxiety Stress Scale
DERS-16: Difficulties in Emotion Regulation Scale
DSHI-Y: Deliberate Self-Harm Inventory
ECR-RC: Experience in Close Relationships
ERITA: Emotion Regulation Individual Therapy for Adolescents
NEQ: Negative Effects Questionnaire
NSSI: Non-suicidal self-injury
RRs: Risk ratios
SDQ: Strengths and Difficulties Questionnaire
WAI-SR: Working Alliance Inventory
