# Supplementary material for: An Internet-based emotion regulation intervention versus no intervention for non-suicidal self-injury in adolescents: a statistical analysis plan for a feasibility randomised clinical trial
Source: Trials. 2021 Jul 16;22:456. doi: 10.1186/s13063-021-05406-2 (PMC8283974; doi:10.1186/s13063-021-05406-2)
Supplement: Supplementary file 1 — Additional file 1. [file 13063_2021_5406_MOESM1_ESM.pdf]

Statistical report for TEENS feasibility trial

Using simulated data

14 juni 2021

Contents

Baseline characteristics 2

Feasibility outcomes 3

Exploratory clinical outcomes 4

DSHI-Y (primary) 4
Kidscreen-10 (secondary) 5
DASS-21 (secondary) 7
Self-injury (secondary) 10
Sick days (secondary) 11
DERS-16 (further) 12
BSL-supplement (further) 13
CCNES-APP (further) 14
NEQ (further) 15

Baseline characteristics

Baseline characteristics

	A	B	Overall
n	15	15	30
Age (mean (SD))	16.13 (0.83)	15.64 (0.50)	15.90 (0.72)
Gender (%)			
Female	6 (40.0)	5 (33.3)	11 (36.7)
Male	3 (20.0)	2 (13.3)	5 (16.7)
Other	3 (20.0)	3 (20.0)	6 (20.0)
Transgender	3 (20.0)	5 (33.3)	8 (26.7)
Nationality (%)			
Danish	4 (26.7)	6 (40.0)	10 (33.3)
Middle Eastern	3 (20.0)	6 (40.0)	9 (30.0)
Other	5 (33.3)	1 (6.7)	6 (20.0)
Other European/North American	3 (20.0)	2 (13.3)	5 (16.7)
School (%)			
Boarding school	1 (6.7)	4 (26.7)	5 (16.7)
High-school	3 (20.0)	3 (20.0)	6 (20.0)
Middle-school	6 (40.0)	3 (20.0)	9 (30.0)
No school	2 (13.3)	2 (13.3)	4 (13.3)
Other	3 (20.0)	3 (20.0)	6 (20.0)
Parental status (%)			
Cohabitant	3 (20.0)	3 (20.0)	6 (20.0)
Divorced	5 (33.3)	5 (33.3)	10 (33.3)
Married	4 (26.7)	2 (13.3)	6 (20.0)
Other	3 (20.0)	5 (33.3)	8 (26.7)

Missing data: Age: 3.3 %

Feasibility outcomes

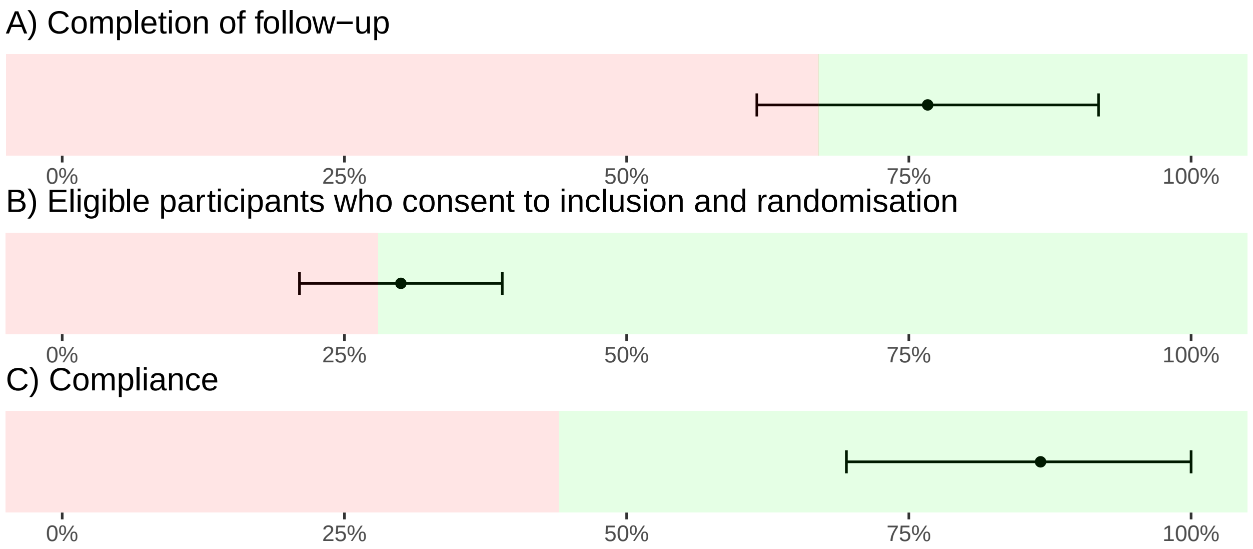

Outcome	Requirement	Fraction	LCL	UCL
A) Completion of follow-up	68%	77%	62%	92%
B) Eligible participants who consent to inclusion and randomisation	29%	30%	21%	39%
C) Compliance	45%	87%	69%	100%

Exploratory clinical outcomes

DSHI-Y (primary)

Data on non-suicidal self-injury (NSSI) as measured by DSHI-Y will be obtained in both groups at baseline and follow-up, and during the intervention for the interventional group. DSHI-Y reflects the number of NSSI events in the past month. The results will be presented as one sum score.

Results

Summarised results

	A<br>mean (SD) [n]	B<br>mean (SD) [n]	Overall
n	15	15	30
DSHI-Y (baseline)	210.93 (126.99) [15]	208.33 (107.99) [15]	209.63 (115.83)
DSHI-Y (follow-up)	183.47 (127.14) [15]	171.00 (134.16) [15]	177.23 (128.58)

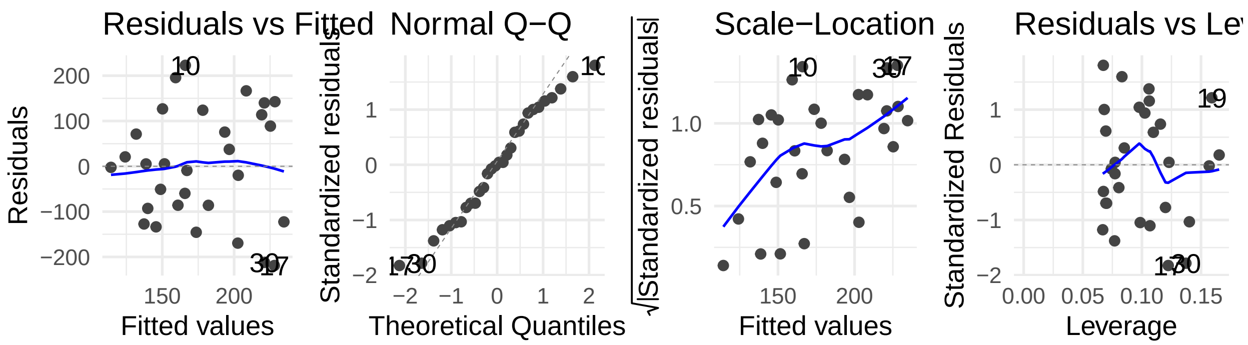

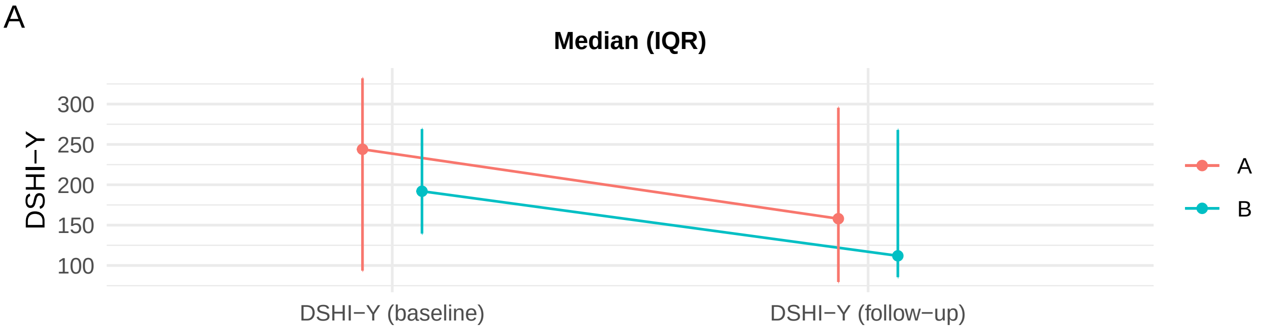

Linear regression

	p-value
DSHI-Y	0.80463

Kidscreen-10 (secondary)

Data on Kidscreen-10 will be obtained in both groups at baseline and follow-up. Kidscreen-10 has 10 items which each generates a score from 1 to 5. The sum of the raw scores will be processed using the algorithm below:

- *RASCH PP* = (10 = -4.327) / (11 = -3.206) / (12 = -2.675) / (13 = -2.319) / (14 = -2.049) / (15 = -1.831) / (16 = -1.645) / (17 = -1.483) / (18 = -1.339) / (19 = -1.207) / (20 = -1.085) / (21 = -0.971) / (22 = -0.863) / (23 = -0.760) / (24 = -0.660) / (25 = -0.563) / (26 = -0.468) / (27 = -0.374) / (28 = -0.281) / (29 = -0.187) / (30 = -0.093) / (31 = 0.002) / (32 = 0.100) / (33 = 0.199) / (34 = 0.302) / (35 = 0.409) / (36 = 0.520) / (37 = 0.637) / (38 = 0.760) / (39 = 0.891) / (40 = 1.031) / (41 = 1.183) / (42 = 1.348) / (43 = 1.529) / (44 = 1.732) / (45 = 1.961) / (46 = 2.226) / (47 = 2.545) / (48 = 2.951) / (49 = 3.533) / (50 = 4.703)

(points = sub-score)

The results are presented using two scales:

- **General HRQoL index** = RASCH PP
- **General HRQoL index European T-values** = (((RASCH PP - 1.2078) / 1.03377) * 10 + 50)

Results

Summarised results

	A<br>mean (SD) [n]	B<br>mean (SD) [n]	Overall
n	15	15	30
Kidscreen-10 T-values (baseline)	62.43 (9.51) [14]	58.19 (12.19) [15]	60.23 (11.00)
Kidscreen-10 T-values (follow-up)	20.45 (11.54) [14]	19.05 (12.83) [15]	19.72 (12.03)

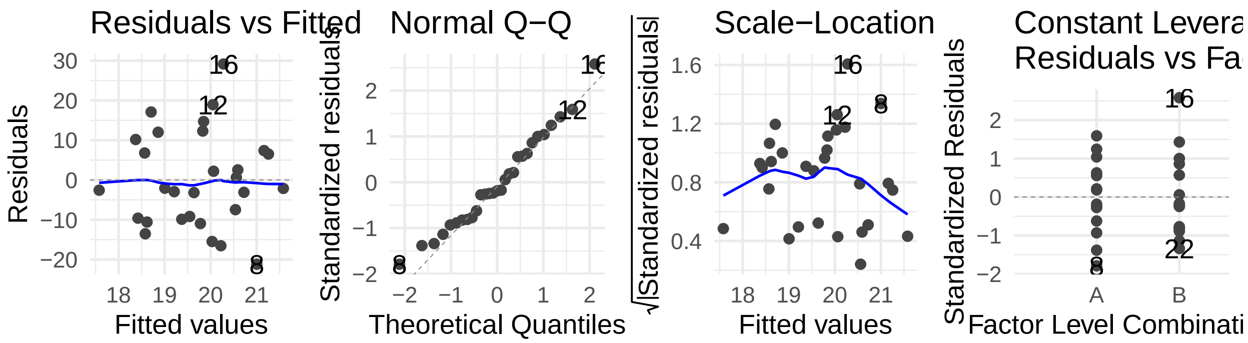

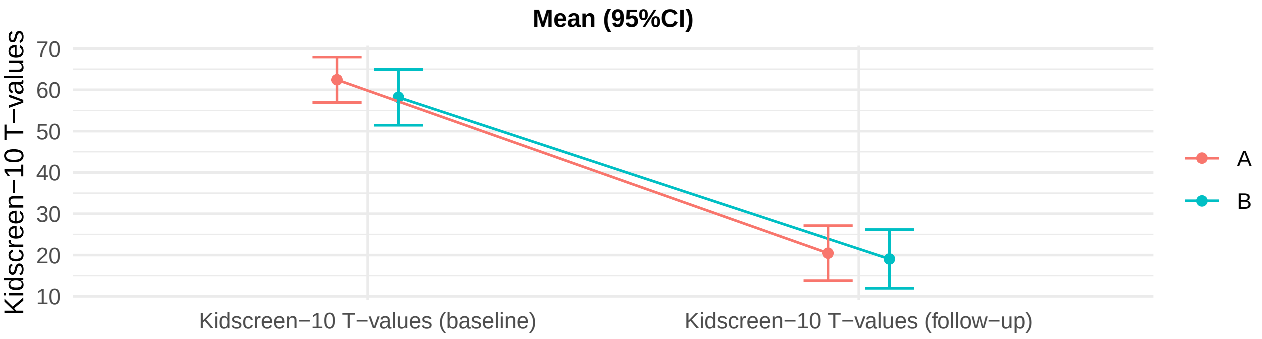

Linear regression

	p-value
Kidscreen-10 T-values	0.72449

DASS-21 (secondary)

Data on DASS-21 will be obtained in both groups at baseline and follow-up. DASS-21 has 21 items which each generates a score from 0 to 3. The results will be presented as sum-score for the following three subscales:

- **Depression** = I3 + I5 + I10 + I13 + I16 + I17 + I21
- **Anxiety** = I2 + I4 + I7 + I9 + I15 + I19 + I20
- **Stress** = I1 + I6 + I8 + I11 + I12 + I14 + I18

I: Items

Results

Summarised results

	A mean (SD) [n]	B mean (SD) [n]	Overall
n	15	15	30
DASS-21 Depression (baseline)	10.00 (6.08) [15]	11.13 (6.57) [15]	10.57 (6.25)
DASS-21 Anxiety (baseline)	8.60 (5.65) [15]	13.27 (5.78) [15]	10.93 (6.10)
DASS-21 Stress (baseline)	11.07 (6.89) [15]	10.87 (5.01) [15]	10.97 (5.92)
DASS-21 Depression (follow-up)	9.00 (6.35) [15]	10.33 (6.50) [15]	9.67 (6.35)
DASS-21 Anxiety (follow-up)	10.20 (7.22) [15]	9.20 (5.94) [15]	9.70 (6.52)
DASS-21 Stress (follow-up)	9.33 (7.83) [15]	10.27 (7.05) [15]	9.80 (7.33)

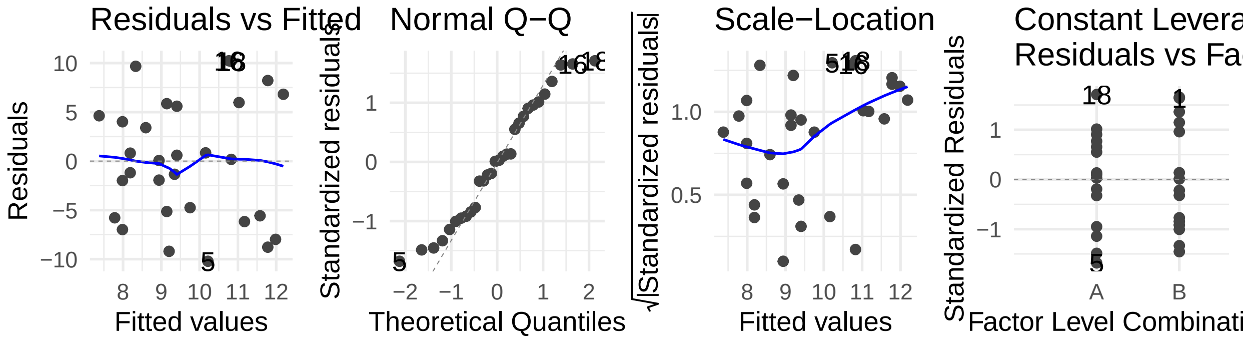

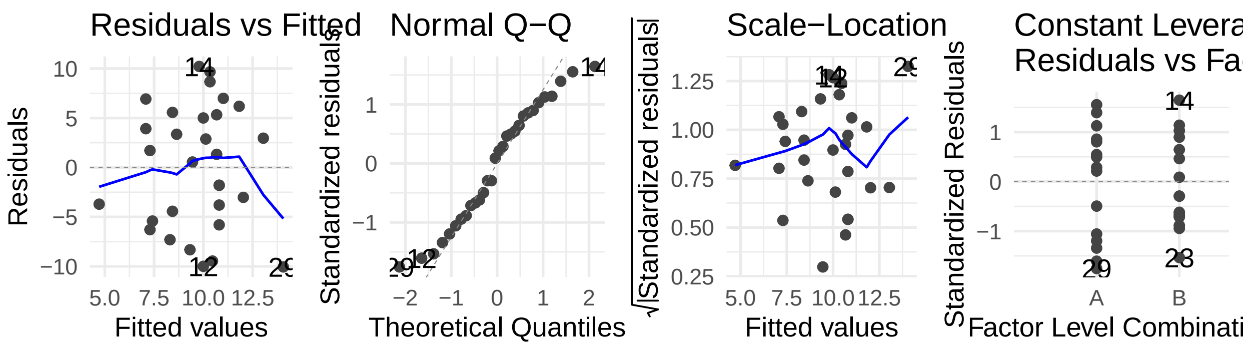
Residuals vs Fitted
Residuals
Fitted values
Normal Q−Q
Standardized residuals
Theoretical Quantiles
Scale−Location
√|Standardized residuals|
Fitted values
Constant Levera
Residuals vs Fa
Standardized Residuals
Factor Level Combinati
A
B

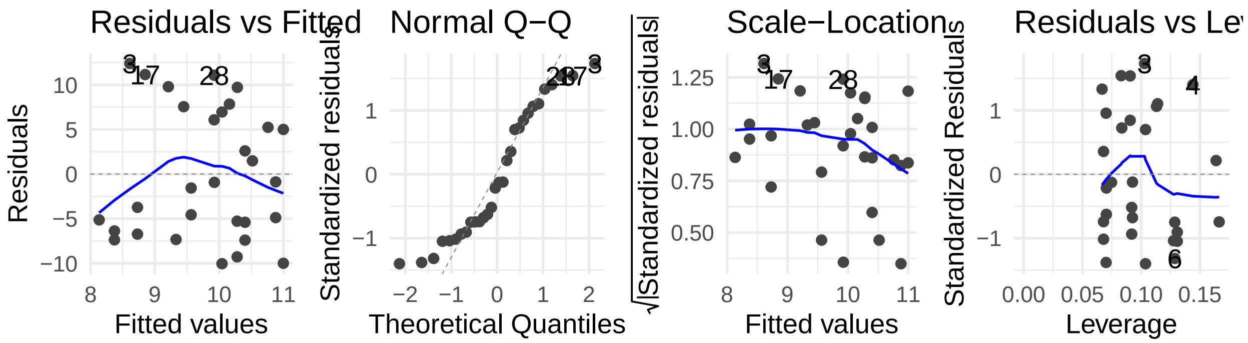
Residuals vs Fitted
Residuals
Fitted values
Normal Q−Q
Standardized residuals
Theoretical Quantiles
Scale−Location
√|Standardized residuals|
Fitted values
Residuals vs Le
Standardized Residuals
Leverage

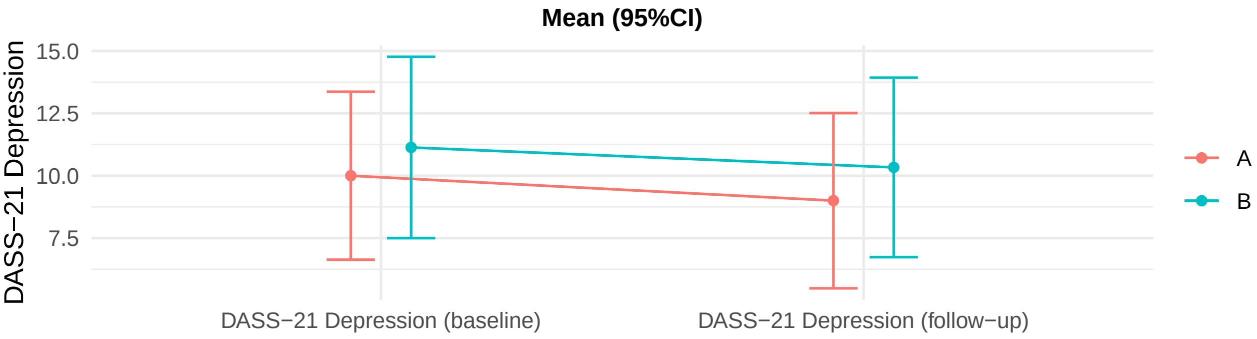
Mean (95%CI)
DASS−21 Depression
DASS−21 Depression (baseline)
DASS−21 Depression (follow−up)
A
B

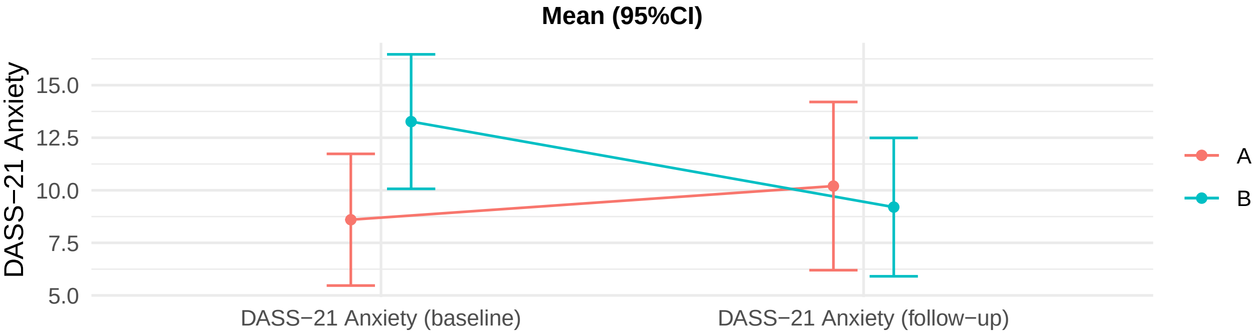
Mean (95%CI)
DASS−21 Anxiety
DASS−21 Anxiety (baseline)
DASS−21 Anxiety (follow−up)
A
B

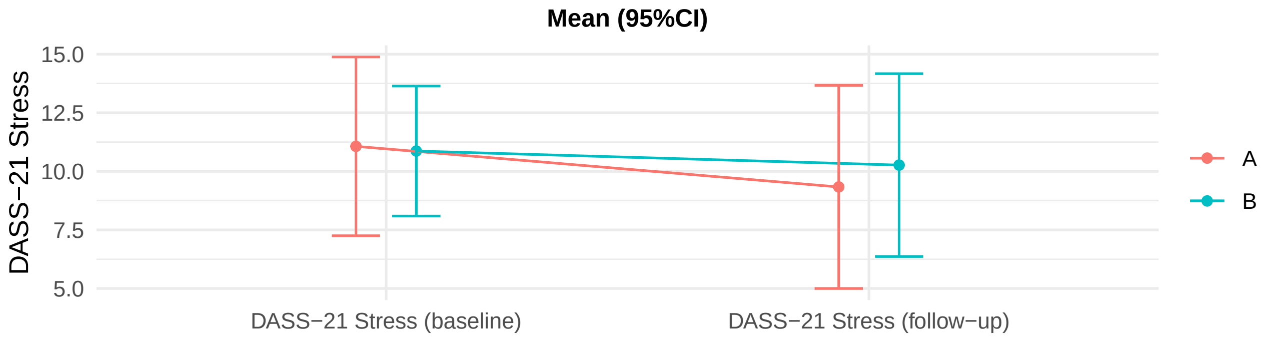

Linear regression

	p-value
DASS-21 Depression	0.51159
DASS-21 Anxiety	0.32147
DASS-21 Stress	0.73107

Self-injury (secondary)

Data on 'Self-injury' wil be obtained in both groups only at follow-up. 'Self-injury' is answered as either 'yes' or 'no'.

Summarised results	Summarised results A N (%) [n]	B N (%) [n]	Summarised results Overall
n	15		30
Self-injury = Yes (%)	8 (53.3) [15]	9 (60.0) [15]	17 (56.7)

var		R	lcl	ucl	p
Self-injury	OR	1.31	0.31	5.75	0.713
Self-injury	RR	1.12	0.60	2.11	

NB: Confidence intervals of RR are *Unconditional MLE & normal approximation (Wald) CI*, while the confidence intervals for RR will be obtained using marginal effects.

Sick days (secondary)

Data on ‘Sick days’ wil be obtained in both groups at baseline and follow-up. ‘Sick days’ will be presented as a ratio, where 0 refers to full student capacity.

Summarised results

	A<br>mean (SD) [n]	B<br>mean (SD) [n]	Overall
n	15	15	30
Sick days (baseline)	0.59 (0.29) [15]	0.44 (0.22) [15]	0.51 (0.26)
Sick days (follow-up)	0.52 (0.22) [15]	0.48 (0.26) [15]	0.50 (0.24)

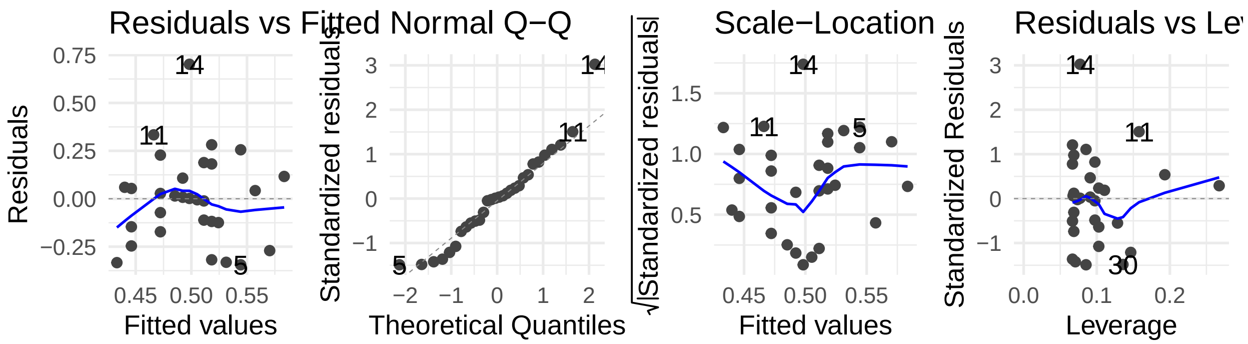

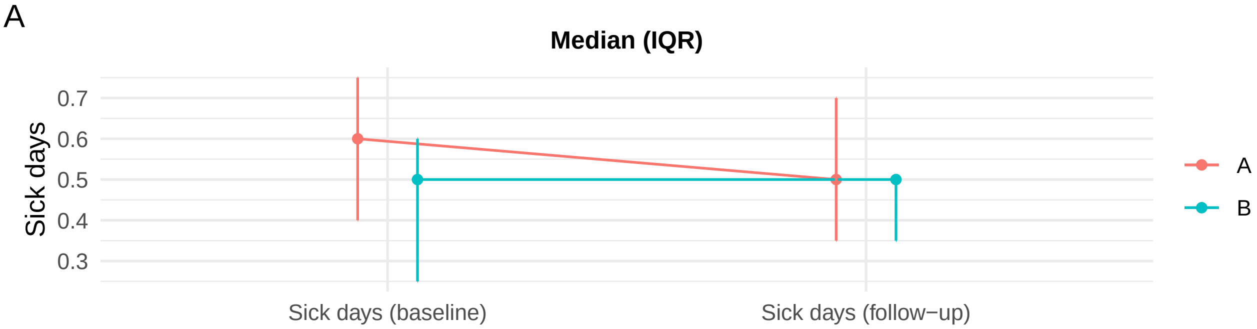

Linear regression

	p-value
Sick days	0.52578

DERS-16 (further)

Data on DERS-16 will be obtained in both groups at baseline and follow-up, and during the intervention for the interventional group. DERS-16 has 16 items which each generates a score from 1 to 5. The results will be presented as one sum-score. The scores in this scale range from 16-80 with higher scores indicating greater emotion regulation difficulties.

Results

Summarised results

	A<br>mean (SD) [n]	B<br>mean (SD) [n]	Overall
n	15	15	30
DERS-16 (baseline)	54.53 (19.25) [15]	48.00 (16.82) [15]	51.27 (18.07)
DERS-16 (follow-up)	37.33 (21.02) [15]	46.40 (18.70) [15]	41.87 (20.09)

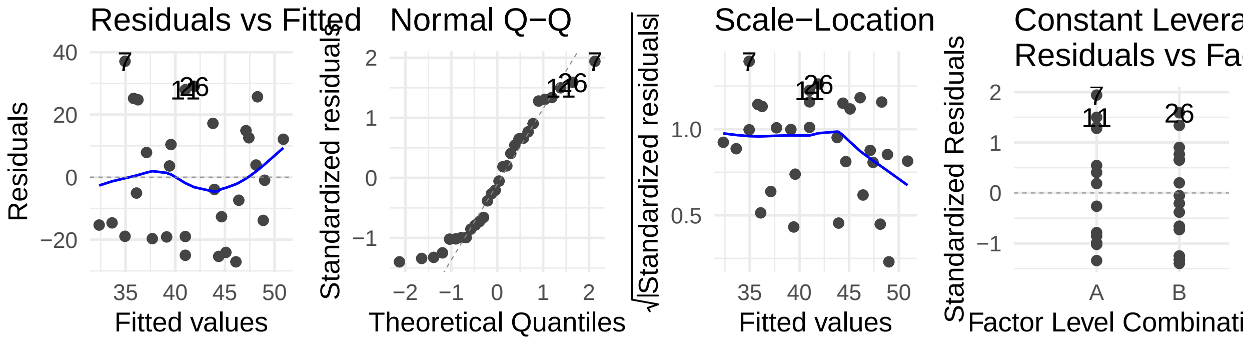

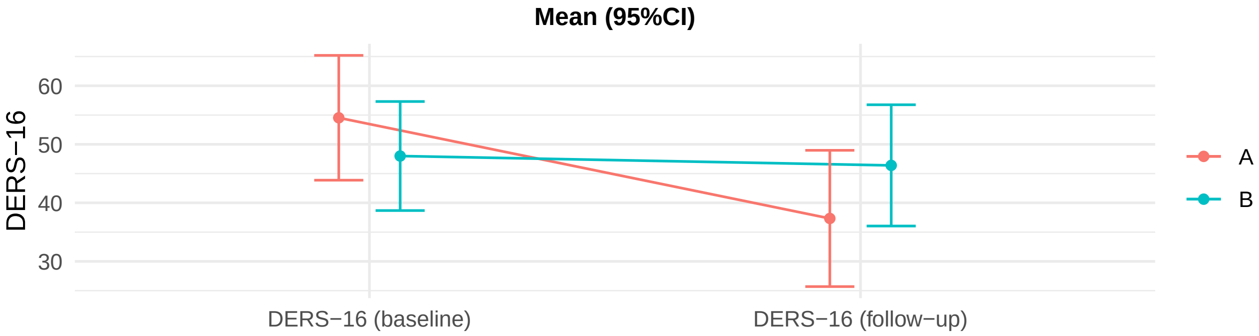

Linear regression

	p-value
DERS-16	0.19082

BSL-supplement (further)

Data on BSL-supplement will be obtained in both groups at baseline and follow-up, and during the intervention for the interventional group. BSL-supplement has 11 items which each generates a score from 0 to 4. The results will be presented as one sum-score.

Results

Summarised results

	A<br>mean (SD) [n]	B<br>mean (SD) [n]	Overall
n	15	15	30
BSL-supplement (baseline)	19.80 (13.99) [15]	24.20 (14.97) [15]	22.00 (14.41)
BSL-supplement (follow-up)	18.60 (14.68) [15]	16.27 (13.08) [15]	17.43 (13.72)

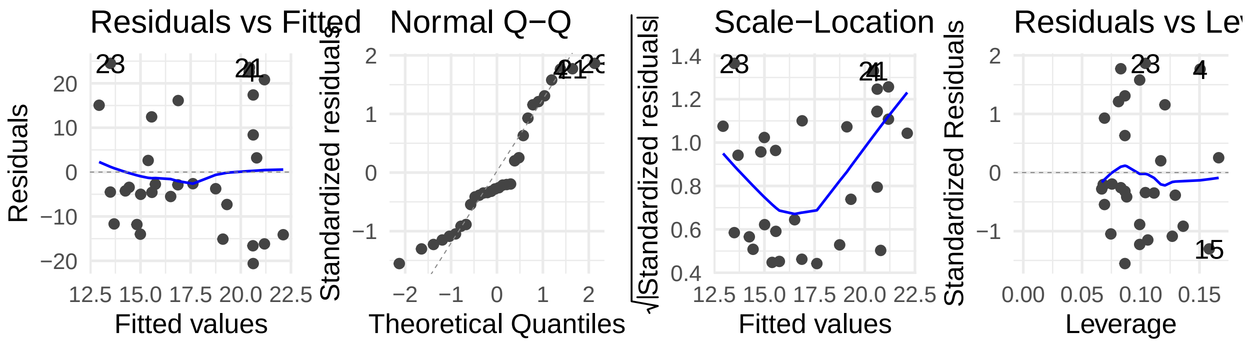

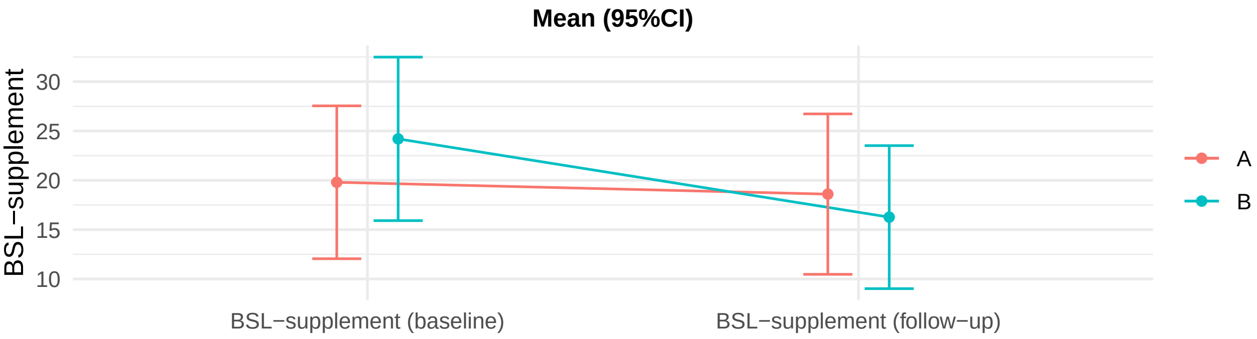

Linear regression

	p-value
BSL-supplement	0.77085

CCNES-APP (further)

Data on CCNES-APP will be obtained from the participants in both groups only at follow-up. CCNES-APP has 9 main items with 6 sub items (a to f) for each. Each sub item generates a score from 1 to 7. The results will be presented as mean-scores for the following six subscales:

- **Distress reaction** = (I1a + I2d + I3f + I4d + I5a + I6b + I7f + I8a + I9d) / 9
- **Punitive reactions** = (I1e + I2f + I3b + I4c + I5e + I6c + I7b + I8e + I9c) / 9
- **Expressive encouragment** = (I1b + I2c + I3e + I4f + I5b + I6d + I7e + I8b + I9e) / 9
- **Emotion-focused reactions** = (I1c + I2b + I3c + I4a + I5c + I6f + I7d + I8c + I9a) / 9
- **Problem-focused reactions** = (I1f + I2e + I3a + I4b + I5f + I6e + I7a + I8f + I9b) / 9
- **Minimization reactions** = (I1d + I2a + I3d + I4e + I5d + I6a + I7c + I8d + I9f) / 9

I: Items

Results

Summarised results

	A mean (SD) [n]	B mean (SD) [n]	Overall
n	15	15	30
CCNES-APP Distress reaction (follow-up)	3.39 (1.06) [15]	3.33 (0.77) [15]	3.36 (0.91)
CCNES-APP Punitive reactions (follow-up)	3.79 (0.85) [15]	4.23 (0.79) [15]	4.01 (0.84)
CCNES-APP Expressive encouragment (follow-up)	3.67 (1.25) [15]	3.91 (1.08) [15]	3.79 (1.16)
CCNES-APP Emotion-focused reactions (follow-up)	3.52 (0.90) [15]	3.87 (1.19) [15]	3.69 (1.05)
CCNES-APP Problem-focused reactions (follow-up)	3.86 (1.28) [15]	3.78 (0.92) [15]	3.82 (1.10)
CCNES-APP Minimization reactions (follow-up)	3.41 (1.20) [15]	3.56 (0.83) [15]	3.49 (1.02)

Mann-Whitney U test

	Estimate (95% Confidence interval)	p-value
Distress reaction	0.3 (-0.4-0.8)	0.3825
Punitive reactions	-0.5 (-1-0.2)	0.2124
Expressive encouragment	-0.2 (-1-0.6)	0.5192
Emotion-focused reactions	-0.3 (-1-0.5)	0.406
Problem-focused reactions	0.2 (-0.9-1.1)	0.7082
Minimization reactions	-0.2 (-0.9-0.6)	0.6629

NEQ (further)

Data on NEQ will be obtained in both groups only at follow-up. NEQ has 20 items, with 5 sub-items The results will be presented as means and standard deviations:

- **Frequency negative effects** = I1a + I2a + I3a + I4a + I5a + I6a + I7a + I8a + I9a + I10a + I11a + I12a + I13a + I14a + I15a + I16a + I17a + I18a + I19a + I20a *(Score from 0 to 1)*

Summarised results

	A<br>mean (SD) [n]	B<br>mean (SD) [n]	Overall
n	15	15	30
NEQ Freq. negative effects	8.40 (6.14) [15]	9.53 (5.59) [15]	8.97 (5.80)

Mann-Whitney U test

	Estimate (95% Confidence interval)	p-value
NEQ Freq. negative effects	-1 (-6-3)	0.5465
